# Supplementary material for: Pathogenic Variants in USH1G/SANS Alter Protein Interaction with Pre-RNA Processing Factors PRPF6 and PRPF31 of the Spliceosome
Source: Int J Mol Sci. 2023 Dec 18;24(24):17608. doi: 10.3390/ijms242417608 (PMC10744108; doi:10.3390/ijms242417608)
Supplement: Supplementary file 1 [file ijms-24-17608-s001.zip › MDPI_IJMS_Supplementary_Material_Fritze_et_al.pdf]

Supplementary Material

Pathogenic Variants in *USH1G*/SANS Alter Protein Interaction with Pre-RNA Processing Factors PRPF6 and PRPF31 of the Spliceosome

Jacques S. Fritze, Felizitas F. Stiehler and Uwe Wolfrum *

Institute of Molecular Physiology, Johannes Gutenberg University Mainz, 55128 Mainz, Germany
* Correspondence: wolfrum@uni-mainz.de

1 Supplementary Figures and Tables

Supplementary_Figure_File.docx: all Figures S1-S8 referred to in this work;
Table S1.xlsx: nucleic acids used in this study;
Table S2.xlsx: contacts of PRPF31-NOP_SANS-CENTn1, contacts of PRPF6-Cterm_SANS-CENTn, contacts of PRPF31-NOP_SANS-ΔHydro.
Table-S3.xlsx: lists of vertebrates and mammals used for analyses of evolutionary conservation
Github code and predictions: https://github.com/LabWolfrum/Fritze_et_al.2023.git

1.1 Supplementary Figures

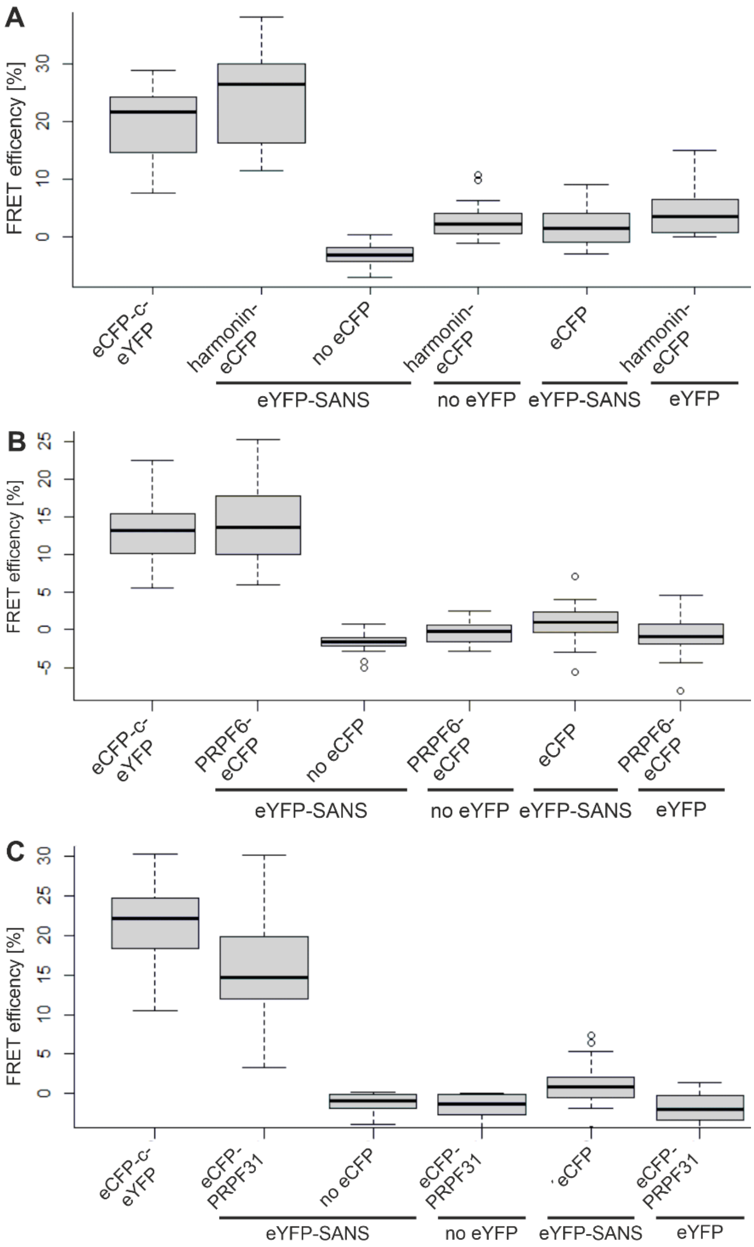

Figure S1. FRET acceptor bleached assays of SANS and binding partners. (A-C) FRET assay with transfected HEK293T cells as indicated. As positive control, eCFP-c-eYFP fusion construct was used. As a negative control, eCFP/eYFP without a fusion protein was used. **(A)** harmonin-eCFP and eYFP-SANS showed high FRET efficiency to each other but not to the negative controls. **(B)** PRPF6-eCFP and eYFP-SANS showed high FRET efficiency to each other but not to the negative controls. **(C)** eCFP-PRPF31 and eYFP-SANS showed high FRET efficiency to each other but not to the negative controls. One representative experiment out of three independent experiments is shown. Outliers are shown as dots above/below the boxplots.

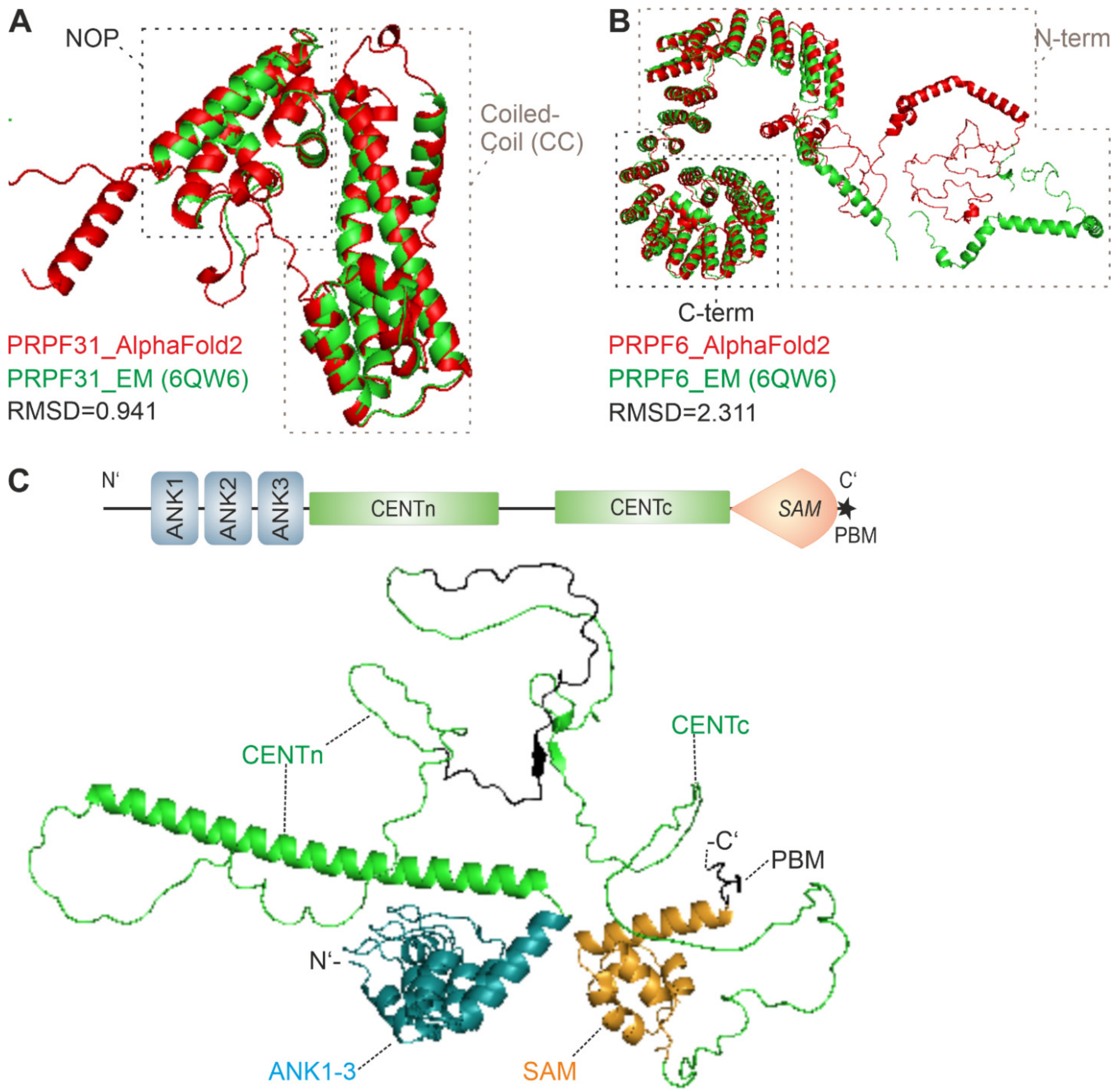

Figure S2. In silico prediction of SANS, PRPF6 and PRPF31 with AlphaFold2. AlphaFold2 predicted structures of PRPF31 **(A)** and PRPF6 **(B)** aligned to the cryoEM structure previously achieved by (Charenton et al., 2019; PDB code: 6QW6). Rout mean square deviation (RMSD) following alignment in PyMol is indicated below the structure. **(C)** Schematic domain structure and AlphaFold2 prediction of SANS. From N- to C-terminal: a structured N-terminus (blue, ankyrin repeats), the CENTn (green) beginning with a long α-helix and ending in an intrinsically disordered region (IDR), the IDR CENTc (green) and structured SAM domain (orange) with IDR of PBM (black).

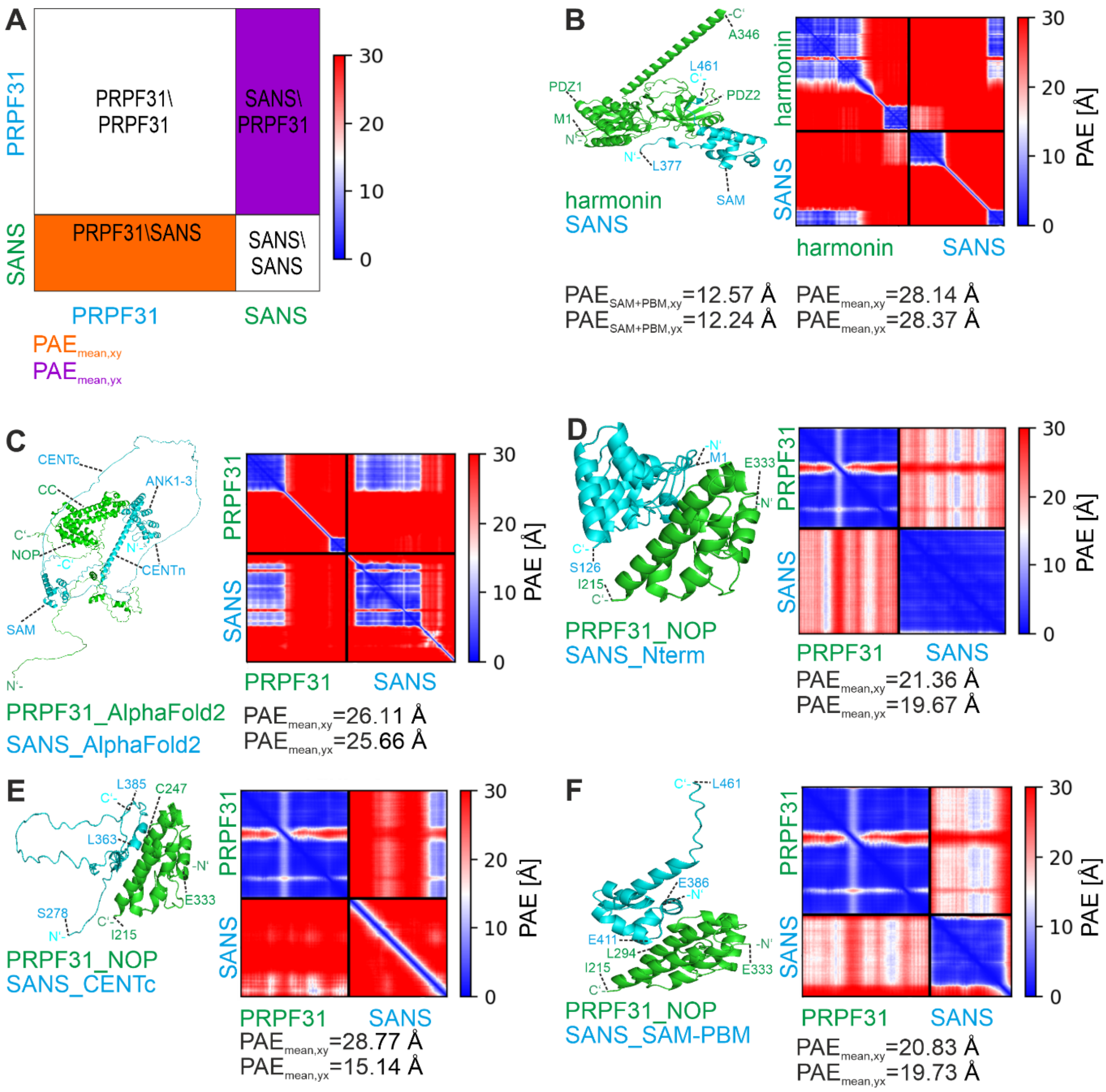

Figure S3. Molecular structure predictions of SANS-harmonin and -PRPF31 complexes with AlphaFold2-multimer. (A) Example predicted alignment error (PAE) of PRPF31 to itself (upper left square) and SANS (lower left square) and SANS to itself (lower right square) and PRPF31 (upper right square). PAE reports AlphaFolds expected position error at residue x when the predicted and true structures are aligned on residue y. A summary of PAE score is calculated for the lower left square ($PAE_{mean,xy}$) and upper right square ($PAE_{mean,yx}$). **(B-F)** *In silico* predictions of SANS-harmonin and PRPF31 complexes by AlphaFold2. **(B)** harmonin (green, aa 1-346) complex with the SANS SAM-PBM domain (blue, aa 377-461). As shown in the PAE diagram, Alphafold2 calculates this complex to be of high confidence in the interacting area. **(C)** Predicted complex of full-length PRPF31 (blue) and SANS (green), which had low confidence shown by PAE diagram. A small part of the N-terminal regions of SANS and PRPF31 were predicted with high confidence. **(D-F)** PRPF31's NOP domain (green, aa 215-333) complex with the N-terminal part of SANS (blue, aa 1-127) **(D)**, the CENTc domain of SANS (blue, aa 278-385) **(E)** and C terminal part of SANS (blue, aa 386-461) **(F)**. All three complexes had a low confidence.

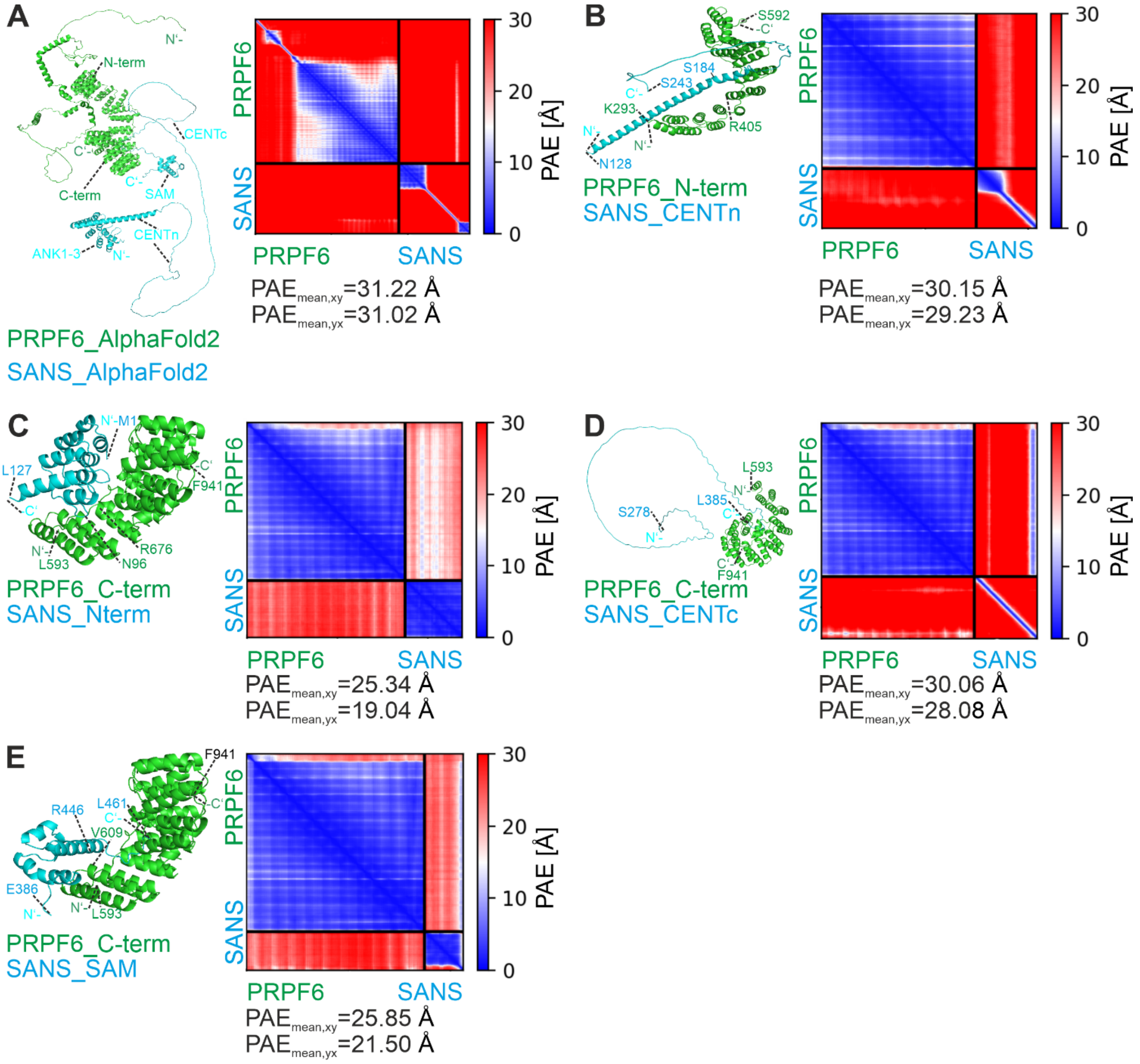

Figure S4. Molecular structure predictions of SANS-PRPF6 complex by AlphaFold2. (A-E) *In silico* predictions of SANS-PRPF6 complexes by AlphaFold2. **(A)** Predicted complex of full-length PRPF6 (green) and SANS (blue), which had low confidence shown by predicted alignment error (PAE) diagram. **(B)** PRPF6 N-term (green, aa 293-592) had no confident prediction with SANS CENTn domain (blue, aa 128-243). **(C)** PRPF6 C-term (green, aa 593-941) had a low confidence prediction with the N-terminus of SANS (blue, aa 1-127) **(C)**, the CENTc domain of SANS (blue, aa 278-385) **(D)** and C-terminal part of SANS (blue, aa 386-461) **(E)**. Summary of PAE_{mean} are high in all structure predictions, indicating low confidence in the interface.

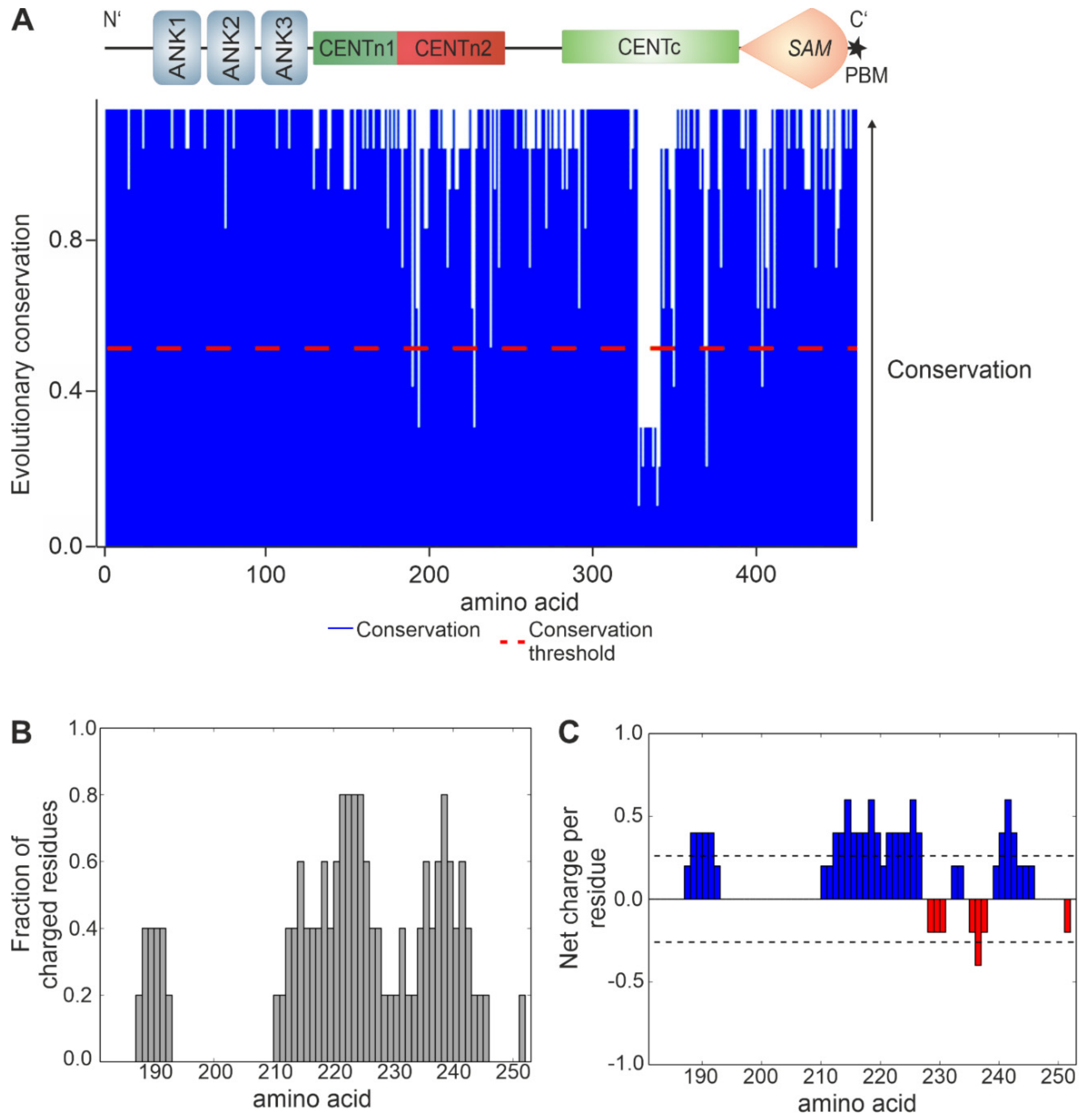

Figure S5. Analyses of the evolutionary conservation of SANS molecules in mammals and analysis of amino acid charges of SANS. (A) Evolutionary conservation (blue) of SANS proteins for 98 mammals. The conservation threshold (red dotted line) indicates high conservation of SANS in mammals except for small regions of CENTn and CENTc domains. **(B)** Fraction of charged residues diagram for SANS CENTn2 predicted by CIDER. Multiple charged residues are prominent between residues 211 to 242. **(C)** Net charge per residue diagram for SANS CENTn2 predicted by CIDER, revealing four loci of positively charged residues (blue).

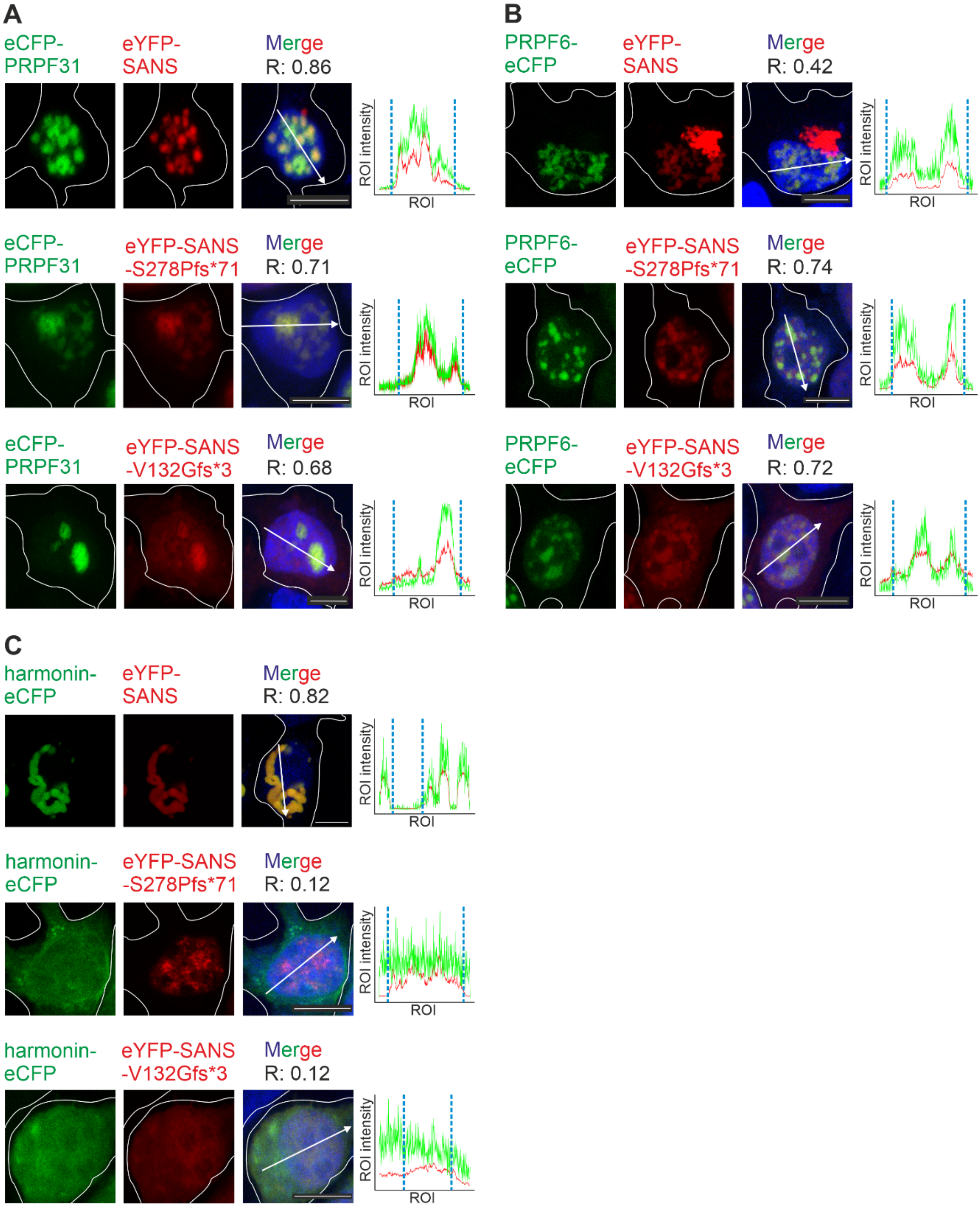
A
eCFP-PRPF31
eYFP-SANS
Merge
R: 0.86
ROI intensity
ROI
eCFP-PRPF31
eYFP-SANS-S278Pfs*71
Merge
R: 0.71
ROI intensity
ROI
eCFP-PRPF31
eYFP-SANS-V132Gfs*3
Merge
R: 0.68
ROI intensity
ROI
B
PRPF6-eCFP
eYFP-SANS
Merge
R: 0.42
ROI intensity
ROI
PRPF6-eCFP
eYFP-SANS-S278Pfs*71
Merge
R: 0.74
ROI intensity
ROI
PRPF6-eCFP
eYFP-SANS-V132Gfs*3
Merge
R: 0.72
ROI intensity
ROI
C
harmonin-eCFP
eYFP-SANS
Merge
R: 0.82
ROI intensity
ROI
harmonin-eCFP
eYFP-SANS-S278Pfs*71
Merge
R: 0.12
ROI intensity
ROI
harmonin-eCFP
eYFP-SANS-V132Gfs*3
Merge
R: 0.12
ROI intensity
ROI

Figure S6. Co-Localization of pathogenic variants of SANS and splicing proteins PRPF6 and PRPF31. (A-C) Confocal microscopy analyses of HEK293T cells co-transfected with eYFP-tagged variants and eCFP tagged PRPFs, counterstained with SPY650-DNA as a nuclear marker. Fluorescence intensity plots of proteins tagged with eYFP (red) and eCFP (green) for regions of interest (ROI) indicated by white arrows in merge images; blue dashed lines indicate nuclear extension. Positive Pearson coefficient R values indicate co-localization. eYFP-SANS and both pathogenic variants eYFP-SANS^{S278Pfs*71} and eYFP-SANS^{V132Gfs*3} co-localized with eCFP-PRPF31 **(A)** and PRPF6-eCFP **(B)** in the nucleus. **(C)** Neither eYFP-SANS^{V132Gfs*3} nor eYFP-SANS^{S278Pfs*71} co-localize with harmonine-eCFP in the cytoplasm. Example images of n=3 experiments, scale bar = 10 µm.

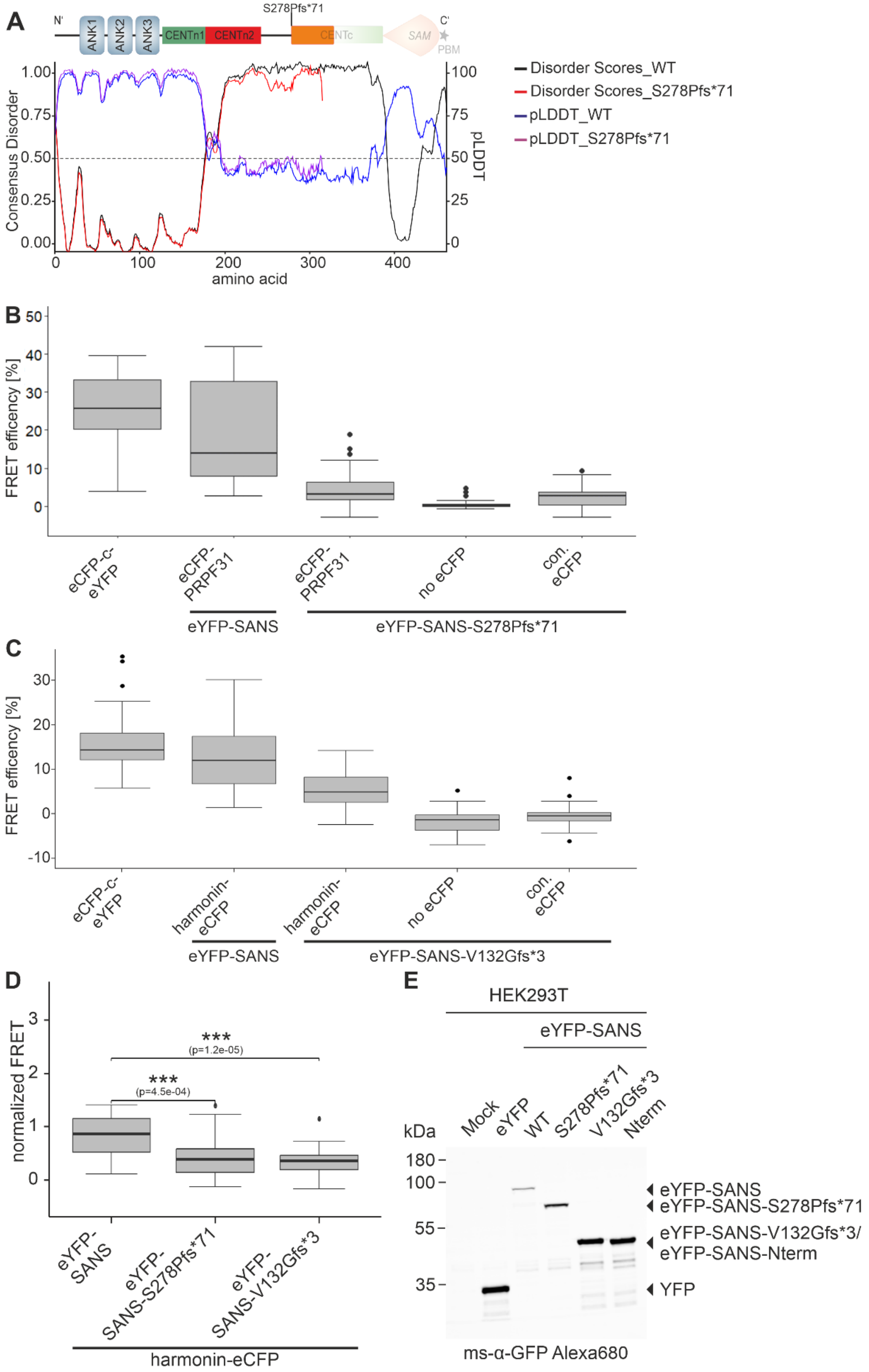

A
N'
ANK1
ANK2
ANK3
CENTn1
CENTn2
S278Pfs*71
CENTc
SAM
C'
PBM
Consensus Disorder
pLDDT
amino acid
Disorder Scores_WT
Disorder Scores_S278Pfs*71
pLDDT_WT
pLDDT_S278Pfs*71
B
FRET efficency [%]
eCFP-c-eYFP
eCFP-PRPF31
eCFP-PRPF31
no eCFP
con. eCFP
eYFP-SANS
eYFP-SANS-S278Pfs*71
C
FRET efficency [%]
eCFP-c-eYFP
harmonin-eCFP
harmonin-eCFP
no eCFP
con. eCFP
eYFP-SANS
eYFP-SANS-V132Gfs*3
D
normalized FRET
***
(p=1.2e-05)
***
(p=4.5e-04)
eYFP-SANS
eYFP-SANS-S278Pfs*71
eYFP-SANS-V132Gfs*3
harmonin-eCFP
E
HEK293T
eYFP-SANS
Mock
eYFP
WT
S278Pfs*71
V132Gfs*3
Nterm
kDa
180
100
55
35
eYFP-SANS
eYFP-SANS-S278Pfs*71
eYFP-SANS-V132Gfs*3/
eYFP-SANS-Nterm
YFP
ms-α-GFP Alexa680

Figure S7. Interaction of pathogenic variants of SANS and binding partners analyzed by FRET acceptor bleach in HEK293T cells. (A) SANS and SANS^{S278Pfs*71} predicted disordered state by Metapredict (black/red line) and AlphaFold2 (blue/purple line). An increase of Metapredict prediction (disordered score) above 0.5 or a decrease in the AlphFold2 prediction (shown by pLDDT) below 50 indicates an intrinsically disordered region (IDR). Metapredict and Alphafold2 predicted no difference between SANS and SANS^{S278Pfs*71}. **(B-D)** FRET assays with eYFP-SANS, eYFP-SANS^{S278Pfs*71} and eYFP-SANS^{V132Gfs*3}paired with eCFP-PRPF31 and harmonin-eCFP **(B-C)** eYFP-SANS^{S278Pfs*71} and -SANS^{V132Gfs*3} showed low FRET efficiency with eCFP-PRPF31 **(B)** and harmonin-eCFP **(C)** similar to multiple negative controls. One representative experiment out of three independent experiments is shown. **(D)** FRET efficiency was normalized to FRET of eCFP-c-eYFP fused tandem pair. Both pathogenic variants SANS^{S278Pfs*71} and SANS^{V132Gfs*3} showed a significant decrease in FRET efficiency to harmonin-eCFP compared to eYFP-SANS. Outliers are shown as dots above/below the boxplots. Wilcoxon signed-rank test was performed for 3 independent experiment. **(E)** Anti-eYFP Western blot analysis of lysates of HEK293T cells expressing different eYFP-SANS constructs. All transfected constructs are detected with the expected size. Expected size in kDa: eYFP ~27kDa, eYFP-SANS ~80 kDa, eYFP-SANS^{S278Pfs*71} ~66 kDa, eYFP-SANS^{V132Gfs*3} ~42 kDa, eYFP-SANS-Nterm ~42 kDa

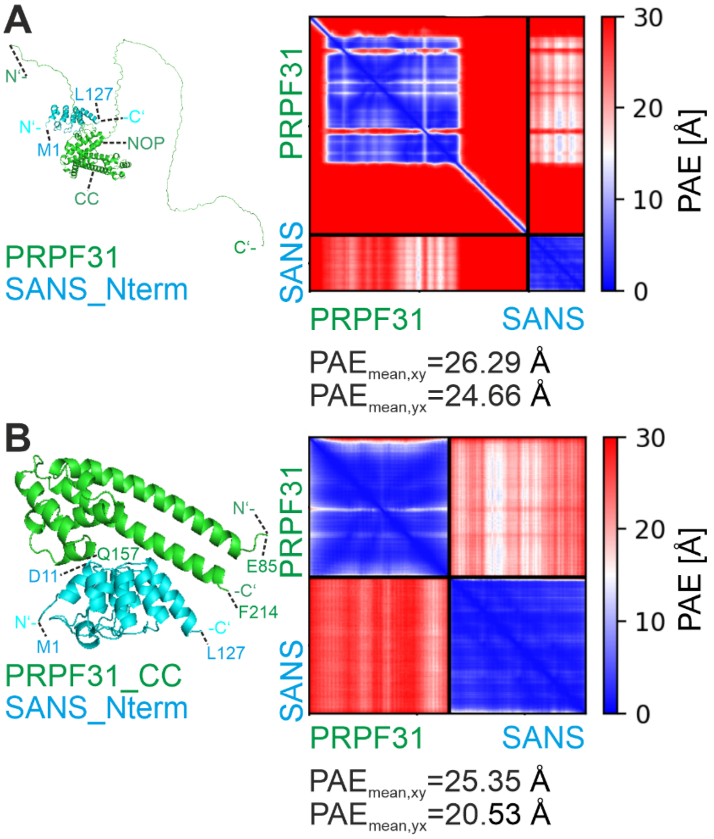

Figure S8. Molecular structure predictions of SANS-PRPF6 complex by AlphaFold2. Predicted complex of full-length PRPF31 (green) and SANS-Nterm (aa 1-127) (blue) **(A),** and of the coiled-coil domain of PRPF31 (aa 85-214) and SANS-Nterm (blue) **(B).** Both predictions had low confidence indicated by predicted alignment error (PAE) diagram and high PAE$_{mean}$.

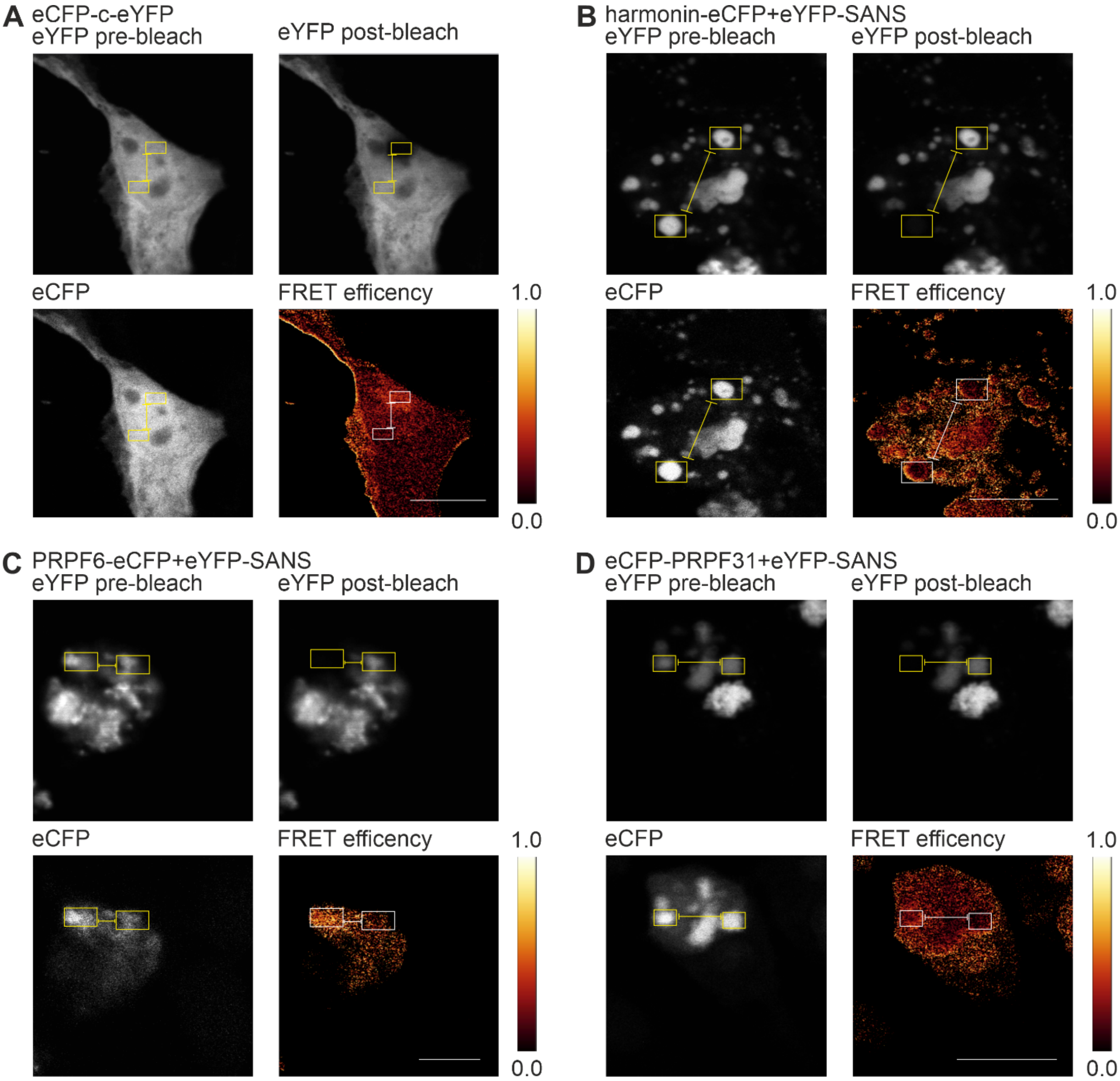

Figure S9. Representative images illustrating FRET acceptor bleaching. (A-D) FRET assay with eYFP/eCFP FRET pairs co-transfected HEK293T cells as indicated. eYFP channel was imaged before (upper left) and after bleaching (upper right). eCFP channel was imaged after bleach of eYFP (lower left). FRET efficiency blot is automatically calculated by LASX. Region of interest (ROI) of bleached and unbleached region (yellow/white square) was used for FRET efficiency calculation. ROIs are a minimum of 3 µm apart.
